# Incidence of dysphagia in acute stroke patients—a prospective multicentre cohort study

**DOI:** 10.3389/fneur.2026.1886254

**Published:** 2026-10-16

**Authors:** Jenny Selg, Vesna Mirkoska, Thorbjörn Holmlund, Levring Jäghagen, Per Wester, Patricia Hägglund

**Affiliations:** 1 Speech and Language Pathology, Department of Clinical Sciences, Faculty of Medicine, Umeå University, Umeå, Sweden; 2 Otorhinolaryngology, Department of Clinical Sciences, Faculty of Medicine, Umeå University, Umeå, Sweden; 3 Oral and Maxillofacial Radiology, Department of Odontology, Faculty of Medicine, Umeå University, Umeå, Sweden; 4 Department of Public Health and Clinical Medicine, Umeå University, Umeå, Sweden; 5 Department of Clinical Sciences, Karolinska Institutet, Danderyd Hospital, Stockholm, Sweden

**Keywords:** acute stroke, deglutition disorder, dysphagia, incidence, oropharyngeal dysphagia, predictors, screening, the Gugging Swallowing Screen

## Abstract

**Background:**

The incidence of dysphagia after stroke remains uncertain in the context of contemporary stroke care. Reported estimates vary widely due to heterogenous assessment methods and limited use of standardized and validated evaluations. Dysphagia is also frequently underestimated despite its strong associations with increased mortality, morbidity, and pneumonia-related hospitalisation. Reliable estimates on incidence based on standardized swallowing assessments are therefore needed to improve identification of dysphagia and to improve clinical management. This study was conducted within the framework of the large prospective multicenter study Dysphagia Evaluation after Stroke (DESIRE) to address these knowledge gaps. Thus, the objective was to determine the incidence of dysphagia in the acute phase following stroke in patients receiving current stroke care using standardized assessment methods, and to identify factors independently associated with post-stroke dysphagia.

**Methods:**

Consecutive acute stroke patients admitted to four Swedish stroke units were assessed within 96 h after stroke onset using the Gugging Swallowing Screen (GUSS) administered by a speech-language pathologist. Clinical data on stroke characteristics, dysphagia risk factors, pneumonia incidence, and discharge destination were prospectively collected. Multivariable regression models were used to identify factors associated with dysphagia.

**Results:**

Of 1,093 patients (468 women, 42.8%; median age 75 years), the incidence of dysphagia was 38.8%. Independent predictors of dysphagia included dysarthria (adjusted odds ratio [aOR]: 1.85, CI = 1.32–2.59, *p* = <0.001), greater stroke severity (aOR: 1.16, CI = 1.12–1.19, *p* = <0.001), female sex (aOR: 1.39, CI = 1.00–1.92, *p =* <0.047), infratentorial lesions (aOR: 2.01, CI = 1.10–3.69, *p* = 0.024) and older age (*p* = <0.001). Dysphagia was significantly associated with longer hospital stay (ratio of mean: 1.64, CI = 1.36–1.98, *p* = <0.001), and with increased odds of discharge to nursing homes (aOR: 4.84, CI = 2.46–9.56, *p* = <0.001), short-term care facilities (aOR: 4.69, CI = 1.87–11.76, *p* = <0.001) or in-hospital death (aOR: 16.67, CI = 2.10–132.05, *p* = 0.008).

**Conclusion:**

Dysphagia affected more than one third of acute stroke patients, and was associated with greater stroke severity, extended hospitalisation, and adverse discharge outcomes. These findings underscore the need for early identification and targeted management of dysphagia to reduce complications and improve patients’ outcomes.

## Background

Swallowing difficulties, commonly referred to as dysphagia, are a frequent complication following stroke. The clinical burden of dysphagia is substantial and is associated with prolonged hospitalisation, increased healthcare utilisation and costs, reduced quality of life (1, 2), and higher mortality among patients with stroke (3, 4).

Up to 50% of stroke patients experience aspiration, defined as entry of saliva, food or liquids into the larynx and below the vocal folds into the trachea (5). Stroke is the leading cause of dysphagia in adults, and the underlying mechanisms and severity of impairment depend on lesion location (6). However, individual differences in brain structure limit the generalizability of research studies, and the ability to predict individual outcomes (7, 8).

Reported incidence estimates of post-stroke dysphagia vary widely, particularly in the acute phase, with published rates ranging from 19 to 81% (2, 4, 9–11). Several factors may explain this heterogeneity. Earlier studies often predate current stroke care, such as acute reperfusion therapy, early mobilisation, and coordinated multidisciplinary management in stroke units (12). In addition, differences in the timing of swallowing assessments relative to stroke onset complicate comparison across studies. More recent investigations are frequently retrospective and rely on non-validated assessment methods, including meta-analyses that aggregate data across heterogeneous study designs, timing of assessments, and definitions of dysphagia (13, 14). These pooled estimates often reflect overall prevalence rather than the proportion of patients who newly develop dysphagia in the acute post-stroke phase and should therefore be interpreted with caution.

Accurate estimation of dysphagia incidence requires use of standardized and validated screening tools that are feasible to apply in large and unselected stroke populations. However, many previous incidence studies have employed screening methods that are not validated against instrumental gold standards such as Videofluoroscopic Swallowing Study (VFSS) or Flexible Endoscopic Evaluation of Swallowing (FEES), or that lack sufficient sensitivity or specificity, increasing the risk of both over- and under-diagnosis (12). The Gugging Swallowing Screen (GUSS) is a step-wise screening instrument that assesses swallowing across multiple consistencies rather than water alone (15). GUSS has demonstrated excellent sensitivity and specificity in comparison with instrumental swallowing assessments, supporting its reliability for identifying dysphagia and aspiration risk in acute stroke populations (16, 17).

Taken together, these limitations highlight a need for prospective studies using standardized and validated swallowing assessments to estimate the incidence of dysphagia in the acute post-stroke phase under current stroke care conditions, and to examine factors associated with its occurrence and clinical consequences.

## Objective

The objective of this study was to determine the incidence of dysphagia in the acute phase following stroke in patients receiving contemporary stroke care and to identify factors independently associated with its occurrence and related clinical consequences.

## Methods

### Design and setting

This study was a prospective observational cohort study involving screening for dysphagia in patients with first-ever stroke in Sweden. The study was conducted as part of the Dysphagia After Stroke (DESIRE) project, aiming to investigate the occurrence of post-stroke dysphagia and associated factors, as well as to evaluate the effects of neuromuscular training in patients with post-stroke dysphagia (clinicaltrial.gov-id: NCT02960737).

Screening for dysphagia among stroke patients was conducted between June 2021 to May 2025. Data collection was initiated at Umeå University Hospital, a tertiary care centre, and subsequently expanded to include two large secondary care hospitals, Danderyd Hospital and Stockholm South General Hospital, and a regional secondary care hospital, Kungälv Hospital.

### Participants

Eligible participants were adults (aged ≥18 years) admitted with a diagnosis of first-ever stroke (ischemic or haemorrhagic). Patients with a previously documented history of dysphagia were excluded. The participants were consecutively recruited from the stroke unit to minimize selection bias.

Written informed consent was obtained from all participants able to provide it. When a participant was physically unable to sign, verbal consent was obtained in accordance with the projects ethical approval. For patients deemed unable to consent due to cognitive impairment, severe aphasia, or lowered level of consciousness, inclusion was permitted using ethically approved proxy procedures to ensure overall cohort coverage.

Swallowing function was primarily assessed using the Gugging Swallowing Screen – Swedish version (GUSS-S) (17) within 96 h after admission. When GUSS-S screening could not be performed within this timeframe due to clinical condition or logistical constraints, a proxy assessment was applied. The proxy consisted of the routinely performed bedside water swallowing assessment, conducted on the stroke unit by the healthcare staff, i.e., registered nurses and assistant nurses, using the Standardized Swallowing Assessment – Swedish version (SSA-S) (18, 19) which served as an indicator of swallowing ability. The use of SSA-S as a proxy measure was justified by previously demonstrated strong correlation between GUSS and SSA-S (16).

This approach ensured near-complete inclusion of eligible patients and reduced the risk of selection bias, as exclusions due to clinical severity and functional status was avoided.

### Procedure

All eligible patients admitted to participating stroke units and neurological intensive care units were systematically screened for post-stroke dysphagia within 96 h of hospital admission. Dysphagia screening was performed using the GUSS-S by trained research speech-language pathologists (*n* = 6), all of whom received standardized instruction in the administration of the assessment.

The GUSS-S evaluates vigilance, saliva management, voluntary cough, and the ability to swallow semi-solid, liquid and solid boluses, administered in a predefined hierarchical order. Total scores range from 0 to 20 and are categorised as no dysphagia (20 points), mild (19–15 points), moderate (14–10 points) or severe dysphagia (9–0 points).

### Variables

The primary outcome was post-stroke dysphagia. Dysphagia was defined as a GUSS score of ≤19 or, when using a proxy outcome, as a failed water swallowing test. Clinical and stroke-related variables were selected based on existing evidence of their association with dysphagia severity, aspiration risk, and clinical outcomes after stroke, and collected from the patients’ medical charts. Stroke severity was assessed using the National Institute of Health Stroke Scale (NIHSS (20)) score, given its established relationship with neurological impairment and dysphagia risk (21, 22). The presence of dysarthria was recorded as a clinical indicator of motor speech impairment following stroke, which may result from lesions affecting various components of the motor speech network (23). Stroke type and lesion location were included due to neuroanatomical involvement within the swallowing network. Hospital-related outcomes were collected to characterise the clinical consequences of dysphagia, including pneumonia during hospitalization (definition based on the treating physician’s assessment using clinical presentation, relevant laboratory findings and radiographic findings when available), tube feeding (i.e., nasogastric (NG) tube, nasoduodenal (ND) tube, nasojejunal (NJ) tube, percutaneous endoscopic gastrostomy (PEG) tube, or radiologically inserted gastrostomy (RIG) tube) occurrence, length of stay and discharge destination. Additionally, potential risk factors for stroke were documented, including obesity (BMI > 30), current smoking, treated hyperlipidaemia, treated hypertension, atrial fibrillation arrhythmia, diabetes mellitus and obstructive sleep apnoea (24).

### Study size

The sample size was determined based on a presumed dysphagia incidence of approximately 42%, derived from a recent systematic review and meta-analysis reporting a pooled prevalence estimate using bedside swallowing assessments comparable to those used in the present study (14). Assuming an incidence of 42%, a sample size of 1,000 to 2,400 participants was estimated to yield an incidence estimate within a margin of error of approximately ±2–3% at a 95% confidence interval, and to provide a 90% power (α = 0.05) to detect clinically relevant associations between dysphagia and selected covariates in multivariable analyses.

### Statistical methods

Incidence of post-stroke dysphagia (yes/no) was reported as percentages. Categorical variables were analysed with chi-squared tests and continuous variables with Mann–Whitney U tests. Multivariable logistic regression estimated adjusted odds ratios (ORs) with 95% confidence intervals for associations between dysphagia and sex, dysarthria, stroke type, tentorium lesion location, age, reperfusion and stroke severity (NIHSS). NIHSS was modelled as a continuous variable. Age showed non-linearity and was modelled with a natural cubic spline (4 degrees of freedom), evaluated by likelihood ratio test. Length of stay (LOS) was analysed using a Gamma generalised linear model with log link. Missing values were minimal except for NIHSS (19%) and LOS (6%), causing sample size to vary by outcome and covariate completeness. *p* < 0.05 was considered significant. Analyses were conducted using SPSS 29 and R 4.3.2.

## Results

### Participants

During the study period, 1,093 stroke patients were included, of which 468 (42.8%) were women. Median age was 75 years. Thrombolysis was performed in 21.1% of patients and thrombectomy (with or without thrombolysis) in 24.8%. Most stroke patients were classified as having a mild stroke according to NIHSS, median score 4 (IQR 0–10, range 0–30). Stroke location distribution was as follows: 721 (66.0%) supratentorial, 104 (9.5%) infratentorial, 84 (7.7%) mixed, and 184 (16.8%) unspecified. In total, 622 (57%) patients were included through screening with the GUSS-S, while an additional 471 (43%) were included via proxy method. The mean time from admission to assessment was 37 h (see Figure 1).

**Figure 1:**
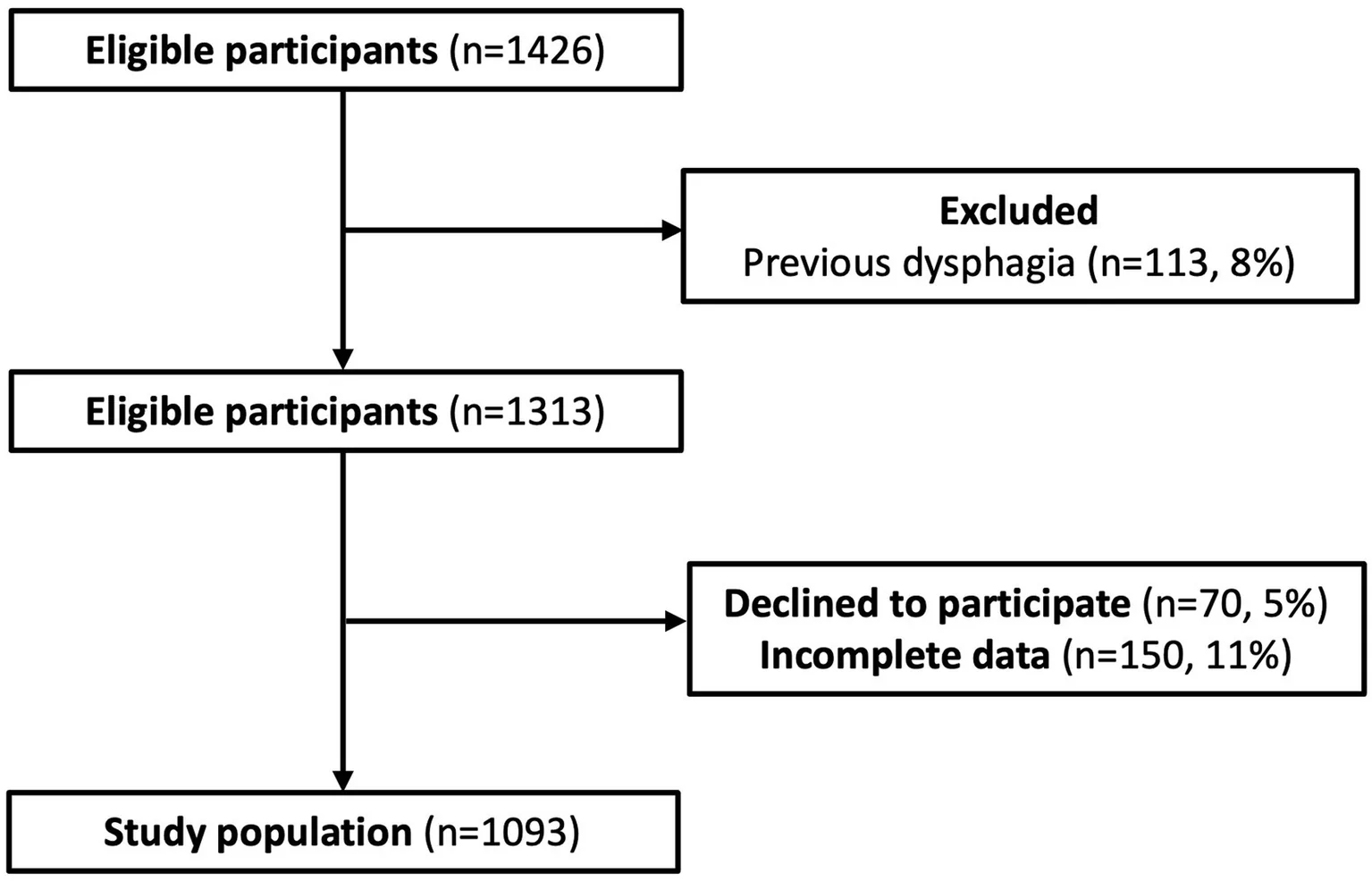
Flowchart showing study inclusion.

### Incidence of dysphagia after stroke

Among first-ever stroke patients, the incidence of dysphagia was 38.8% (see Table 1).

**Table 1:** Baseline characteristics of the total sample stratified by normal swallowing and dysphagia.

Variables	Baseline (*n* = 1,093)	Normal swallowing (*n* = 671)	Dysphagia (*n* = 424)	*p*-value
Age median [IQR]	75 [64.0–82.0]	74 [62.0–81.0]	77 [68.0–84.0]	<0.001
Male sex (%)	625 (57.2)	417 (62.3)	208 (49.1)	<0.001
BMI median* [IQR]	25.7 [23.1–28.6]	25.8 [23.5–28.7]	25.3 [22.2–28.4]	0.058
Obesitas* (%)	133 (12.2)	88 (13.2)	45 (10.6)	0.211
NIHSS median**	4 [1.0–10.0]	2 [0.0–5.0]	4 [4.0–17.0]	<0.001
Stroke type				
Ischemic	869 (79.5)	569 (85.1)	300 (70.8)	<0.001
Haemorrhagic	200 (18.3)	88 (13.2)	112 (26.4)	
Mixed	24 (2.2)	12 (1.8)	12 (2.8)	
Hypertension (%)	621 (56.8)	378 (56.5)	243 (57.3)	0.792
Diabetes mellitus (%)	226 (20.7)	135 (20.2)	91 (21.5)	0.610
Arrhythmia (%)	227 (20.8)	121 (18.1)	106 (25.0)	0.006
Hyperlipidaemia (%)	231 (21.1)	151 (22.6)	80 (18.9)	0.144
OSA (%)	38 (3.5)	25 (3.7)	13 (31.1)	0.555
Smoking (%)	106 (9.7)	72 (10.8)	34 (8.0)	0.135
Lesion location (%)				
Supratentorial	721 (66.0)	424 (63.4)	297 (70.0)	<0.001
Left	347 (48.1)	210 (47.4)	137 (46.1)	
Right	313 (43.4)	182 (42.9)	131 (44.1)	
Mixed	61 (8.5)	32 (7.5)	29 (9.8)	
Infratentorial (%)	104 (9.5)	64 (9.6)	39 (9.2)	
Brain stem involvement	62 (59.6)	37 (57.8)	25 (64.1)	0.139
Mixed	84 (7.7)	44 (6.6)	40 (9.4)	
Unspecified	184 (16.8)	137 (20.5)	48 (11.3)	
Dysarthria (%)	401 (36.7)	196 (29.3)	205 (48.3)	<0.001

Data are presented as *n* (%) or median [IQR]. Statistical analysis was performed using the chi-squared test for categorical variables and the Mann–Whitney U test for continuous variables. BMI, body-mass index; NIHSS, National Institutes of Health Stroke Scale; OSA, obstructive sleep apnoea.

*n = 750; **n = 884.

### Potential predictors of post-stroke dysphagia

Predictors are presented in Table 2. In multivariable analyses, older age (p = <0.001), female sex (adjusted OR [aOR]:1.39, CI = 1.00–1.92, *p =* <0.047), infratentorial lesion location (aOR:2.01, CI = 1.10–3.69, *p* = 0.024), greater stroke severity (NIHSS; aOR:1.16 per point, CI = 1.12–1.19, *p =* <0.001) and dysarthria (aOR:1.85, CI = 1.32–2.59, *p =* <0.001) were independently associated with dysphagia.

**Table 2:** Associated factors (potential predictors) of dysphagia in acute stroke patients.

Variables	OR (95% confidence interval)			
	Unadjusted^a^	*p* value	Adjusted^b^	*P* value
Age (natural cubic spline, df = 4)	—	<0.001	—	<0.001*
Sex (ref. female)	0.60 (0.46–0.80)	<0.001	1.71 (0.51–0.99)	0.045
Stroke type (ref. ischaemic)				
Hemorrhagic	1.67 (1.05–2.63)	0.028	1.65 (0.96–2.81)	0.066
Mixed	2.69 (1.02–7.48)	0.047	1.99 (0.57–6.90)	0.276
Lesion location (ref. supratentorial)				
Infratentorial	0.78 (0.46–1.29)	0.335	1.96 (1.06–3.56)	0.028
Mixed	1.06 (0.61–1.82)	0.826	1.77 (0.91–3.35)	0.085
Unspecified	0.55 (0.37–0.79)	0.002	0.83 (0.52–1.29)	0.400
NIHSS (per point increase)	1.18 (1.15–1.21)	<0.001	1.16 (1.13–1.19)	<0.001*
Dysarthria (ref. normal speech)	1.18 (1.15–1.21)	<0.001	1.87 (1.34–2.61)	<0.001*

Statistical significance ^*^p < 0.001. Age modelled using a natural cubic spline, df = 4; OR not reported. Reference categories: female sex, ischaemic stroke, unspecified lesion location, normal speech. OR, odds ratio; df, degrees of freedom; NIHSS, National Institutes of Health Stroke Scale.

^a^Univariable model; ^b^Multivariable model.

### Potential consequences of post-stroke dysphagia

Consequences of post-stroke dysphagia are presented in Tables 3, 4. Dysphagia was associated with tube feeding (aOR:7.41, CI = 1.55–35.39, *p =* <0.012), longer length of stay (ratio of mean:1.64, CI = 1.36–1.98, *p =* <0.001), increased odds of non-home discharge - hospital transfer (aOR:3.18, CI = 2.01–5.02, *p =* <0.001), ward transfer (aOR:2.23, CI = 1.39–3.57, *p =* <0.001), nursing home (aOR: 4.84, CI = 2.46–9.56, *p =* <0.001) and short-term care facilities (aOR:4.69, CI = 1.87–11.76, *p =* <0.001) as well as in-hospital death (aOR:16.67, CI = 2.10–132.05, *p* = <0.008). Sensitivity analyses using Firth penalised logistic regression showed similar results.

**Table 3:** Associated outcomes in the total sample stratified by normal swallowing and dysphagia.

Variables	Baseline (*n* = 1,093)	Normal swallowing (*n* = 669)	Dysphagia (*n* = 424)	*p*-value
Length of stay median* [IQR]	3 [2–7]	3 [1–5]	5 [2–12]	<0.001
Pneumonia	60 (5.1)	14 (2.0)	46 (10.6)	<0.001
Form of discharge**				<0.001
To home	490 (46.8)	401 (63.4)	89 (21.5)	
Hospital transfer	239 (22.8)	98 (15.5)	141 (34.1)	
Ward transfer	182 (17.4)	98 (15.5)	86 (20.3)	
Nursing home	62 (5.9)	19 (3.0)	43 (10.4)	
Short-term care facility	32 (3.1)	18 (2.6)	18 (4.3)	
Deceased	41 (3.9)	2 (0.3)	39 (9.4)	
Tube feeding***	31 (3.9)	3 (0.6)	28 (8.3)	<0.001

Data are presented as *n* (%) or median number of days [IQR]. Statistical analysis was performed using the chi-squared test for categorical variables and the Mann–Whitney U-test for continuous variables. *n = 1,075, **n = 1,046, ***n = 781.

**Table 4:** Outcomes (potential consequences) of dysphagia in acute stroke patients.

Variables	Effects (95% confidence interval)			
	Unadjusted ^a^	*p* value	Adjusted ^b^	*P* value
Length of stay (in days)	1.82 (1.55–2.16)	<0.001	1.64 (1.36–1.98)	<0.001*
Pneumonia (ref no pneumonia)	3.88 (1.87–8.07)	<0.001	2.45 (1.07–5.63)	0.035
Form of discharge (ref home)				
Hospital transfer	6.03 (4.05–8.96)	<0.001	3.18 (2.01–5.02)	<0.001*
Ward transfer	3.33 (2.22–4.98)	<0.001	2.23 (1.39–3.57)	<0.001*
Nursing home	9.50 (5.07–17.81)	<0.001	4.84 (2.46–9.56)	<0.001*
Short-term care facility	8.48 (3.64–19.74)	<0.001	4.69 (1.87–11.76)	<0.001*
Deceased (ref not deceased)	40.04 (5.37–298.60)	<0.001	16.67 (2.10–132.05)	0.008
Tube feeding (ref no tube feeding)	14.74 (3.37–64.54)	<0.001	7.41 (1.55–35.39)	0.012
Tube feeding (Firth penalised logistic)	12.15 (3.76–61.40)	<0.001	6.01 (1.67–32.16)	0.005

Statistical significance ^*^p < 0.001. Length of stay: Gamma, log; mean ratios. Others; logistic regression, odds ratios. References: no pneumonia, home discharge, no tube feeding.

^a^Univariable model.

^b^Multivariable model, adjusted for age (natural cubic spline, df = 4), sex, NIHSS, and centre.

### GUSS-S versus proxy method

There was a significant difference between those assessed with the GUSS-S and those assessed via the proxy method regarding dysphagia when performing chi-square test (p = <0.001). In the proxy group, 47% had dysphagia compared to 32% in the GUSS-S group.

Among recorded patient characteristics, chi-square revealed two variables that had a significant difference between groups; stroke type (with 29% being hemorrhagic in the proxy group versus 10% in the GUSS-S group, *p* = <0.001) and hyperlipidemia (with 14% in the proxy group and 26% in the GUSS-S group, *p* = <0.001).

Mann–Whitney U test for continuous variables showed a significant difference in NIHSS score, with median 3 in the GUSS-S group and 6 in the proxy group (*p* = <0.001) but not in age or BMI.

## Discussion

This prospective, multicentre study investigated the incidence of dysphagia in patients with first-ever stroke assessed within 96 h of admission and sought to identify risk factors and clinical outcomes. Consecutive patients admitted to stroke units at four Swedish hospitals were systematically screened with validated tools, and clinical data was collected in conjunction with screening. The main finding was a dysphagia incidence of 38.8%.

Comparison of dysphagia incidence across studies is difficult due to methodological heterogeneity, including differences in study design, patient selection, lesion characteristics assessment methods and timing of evaluation. To contextualize our findings, we refer primarily to literature reviews and meta-analyses. In a review published more than two decades ago, Martino et al. reported the dysphagia incidence rates ranging from 37 to 45% when screening-based assessments were used, and from 64 to 78% when instrumental methods were applied (11). Lower incidence estimates were primarily observed in studies restricted to hemispheric stroke populations.

The incidence observed in the present study falls within the same range as the screening-based estimates reported by Martino et al., despite substantial advances in acute stroke care, organisations of stroke services, and early rehabilitation over the past two decades. This finding suggests that, although stroke management has improved, the early incidence of post-stroke dysphagia remains largely unchanged. As in previous studies, lesion location is likely to contribute to variability in reported incidence; brainstem strokes have consistently been associated with higher rates (51–55%) than hemispheric strokes alone (39–40%) (11). Our inclusion of all lesion locations and a larger sample size than most studies included in Martino et al.’s review, strengthens the robustness and contemporary relevance of our findings.

A recent review by Banda et al. (14) found a pooled prevalence of 42% post-stroke dysphagia, closely aligning with the incidence observed in our study (38.8%) (15). However, further comparisons are difficult because the assessments in that review were prevalence rather than incidence, and there was a substantial heterogeneity in assessment timing. Our assessments were conducted within 96 h, whereas other studies varied in timing or lacked timing details. The review also included recurrent strokes, which were associated with a 1.4-fold higher risk of dysphagia (15). Our study focused solely on first-ever stroke, which may have contributed to a slightly lower observed incidence. Overall, these comparisons highlight the need for standardized protocols to improve cross-study interpretability.

Identifying predictors of post-stroke dysphagia is crucial for early clinical management. In this study, dysphagia was more frequently observed in women, aligning with some previous findings (25, 26), though evidence in the literature remains mixed (27, 28). The higher risk observed in women may partly relate to poorer functional outcomes and older age at stroke onset (29). Increasing age was independently associated with dysphagia in our cohort, which is in line with established age-related changes in swallowing physiology and neuromuscular control (30, 31). Greater stroke severity according to NIHSS was also associated with increased risk of dysphagia, coherent with previous research (4, 14, 32). This association is likely to reflect more extensive neurological impairment and greater involvement of cortical and subcortical swallowing networks. Dysarthria emerged as another significant predictor, remaining so in the adjusted analyses. Given the shared muscular and neurological aetiology of dysarthria and dysphagia (33), the former may serve as a clinical indicator of the latter. Data on reperfusion therapy (thrombolysis, thrombectomy, or both) included in the multivariate analysis were available only from Umeå University Hospital and was not independently associated with dysphagia. It is nevertheless notable that the reperfusion rate was high (41.2%). This likely reflects the fact that Umeå University Hospital serves as the comprehensive stroke centre for the whole of northern Sweden, so that patients with proximal large-vessel occlusion who are potentially eligible for thrombectomy are referred there from 11 county hospitals. In univariable analysis, reperfusion therapy was associated with dysphagia, which was more common among patients who received reperfusion (47.6% vs. 36.4%, *p* = 0.002). This is consistent with previous reports; one recent study found an incidence of dysphagia of up to 90% within the first 24 h after thrombectomy (34).

Beyond impaired swallowing, post-stroke dysphagia was associated with adverse clinical outcomes, including significantly longer hospital length of stay. Patients with dysphagia remained hospitalized on average 1.64 times longer than those without dysphagia (95% CI: 1.36–1.98, *p* = <0.001). This aligns with previous research linking dysphagia to extended hospitalization (35), often due to greater sequelae and co-morbidity in dysphagic patients, including respiratory infections (36).

Dysphagia was also associated with increased care dependency (37). Patients with dysphagia were less likely to be discharged directly home and more frequently discharged to other care facilities, reflecting greater post-acute care needs. Transfers to other hospitals were also common in this cohort, likely related to the study’s main centre functioning as a comprehensive stroke and thrombectomy unit, with routine repatriation of patients to referring hospitals. In-hospital mortality was markedly higher among patients with dysphagia, with a 16.7-fold increase in odds, aligning with earlier findings (37).

Tube feeding was often required in patients with severe dysphagia and increased care needs, and was recorded in 3.9% of the cohort. This proportion is lower than previously reported estimates, ranging from 8.5 to 30% (38–40), suggesting a possible underestimation in our study. As tube feeding was identified solely on registered procedure codes, incomplete reporting could be an explanation. Nevertheless, the association between dysphagia and tube feeding remained statistically significant, reinforcing its clinical relevance despite possible underreporting.

A well-recognised complication of dysphagia is pneumonia, that occurred in 5.1% of the cohort, and was independently associated with dysphagia, despite its low occurrence (36, 41). Restricting the analysis to confirmed diagnoses may have underestimated the incidence. Improvements in stroke care, including early mobilisation and routine dysphagia screening, may also have contributed to lower pneumonia rates in acute stroke patients (42). However, approximately one in 10 patients with dysphagia developed pneumonia, underscoring the continued need for effective preventive strategies.

Overall, post-stroke dysphagia remains a significant challenge in current stroke care. Its associations with prolonged hospitalisation, increased likelihood of non-home discharge, higher mortality, and greater need for tube feeding, indicate a substantial clinical and healthcare burden. These findings are consistent with previous studies demonstrating increased direct medical costs and broader societal costs associated with post-stroke dysphagia (43, 44).

Strengths of this study include its prospective design and the use of standardized measures of swallowing ability, both of which are uncommon at this scale within the field. The multicentre design further enhances the generalisability of the findings. Given that reduced vigilance is common in the acute post-stroke period, a proxy screening method was employed to minimize sampling bias and to ensure the inclusion of a broad and clinically representative cohort. Several limitations should also be acknowledged. For participants assessed with the GUSS-S, blinding of assessors was not feasible, as review of medical records was required to verify eligibility. This process inevitably exposed clinical information related to swallowing function, such as prior screening results and current nutritional status, which may have introduced observer bias. In addition, swallowing function was assessed using screening tools rather than instrumental methods. Use of VFSS or FEES would likely have identified a higher incidence of dysphagia due to their greater diagnostic sensitivity (17). However, implementation of instrumental assessments would not have been feasible, given the large cohort size. Information on some variables (NIHSS, reperfusion therapy, BMI) were not available for the whole cohort which may influence the analyses and statistical power of the results.

## Conclusion

In this prospective multicentre cohort study, post-stroke dysphagia was found in 38.8% of the patients with first-ever stroke. Older age, female sex, greater stroke severity, and the presence of dysarthria were independently associated with dysphagia. Dysphagia was further associated with clinically important outcomes, including prolonged hospitalisation, tube feeding, and discharge to settings other than home. These findings underscore the need for early identification and targeted management of dysphagia to mitigate adverse clinical outcomes.

## Data Availability

The raw data supporting the conclusions of this article will be made available by the last author PH, patricia.hagglund@umu.se.
